# Structural Analysis of a Nitrogenase Iron Protein from Methanosarcina acetivorans: Implications for CO_2_ Capture by a Surface-Exposed [Fe_4_S_4_] Cluster

**DOI:** 10.1128/mBio.01497-19

**Published:** 2019-07-09

**Authors:** Lee A. Rettberg, Wonchull Kang, Martin T. Stiebritz, Caleb J. Hiller, Chi Chung Lee, Jasper Liedtke, Markus W. Ribbe, Yilin Hu

**Affiliations:** a Department of Molecular Biology and Biochemistry, University of California, Irvine, California, USA; b Department of Chemistry, University of California, Irvine, California, USA; University of Massachusetts Amherst; University of California, Riverside, USA; University of Basel, Switzerland

**Keywords:** CO_2_ capture, FeS cluster, iron protein, methanogen, nitrogenase

## Abstract

Nitrogenase iron (Fe) proteins reduce CO_2_ to CO and/or hydrocarbons under ambient conditions. Here, we report a 2.4-Å crystal structure of the Fe protein from Methanosarcina acetivorans (*Ma*NifH), which is generated in the presence of a reductant, dithionite, and an alternative CO_2_ source, bicarbonate. Structural analysis of this methanogen Fe protein species suggests that CO_2_ is possibly captured in an unactivated, linear conformation near the [Fe_4_S_4_] cluster of *Ma*NifH by a conserved arginine (Arg) pair in a concerted and, possibly, asymmetric manner. Density functional theory calculations and mutational analyses provide further support for the capture of CO_2_ on *Ma*NifH while suggesting a possible role of Arg in the initial coordination of CO_2_ via hydrogen bonding and electrostatic interactions. These results provide a useful framework for further mechanistic investigations of CO_2_ activation by a surface-exposed [Fe_4_S_4_] cluster, which may facilitate future development of FeS catalysts for ambient conversion of CO_2_ into valuable chemical commodities.

## Abstract (precis)

This work reports the crystal structure of a previously uncharacterized Fe protein from a methanogenic organism, which provides important insights into the structural properties of the less-characterized, yet highly interesting archaeal nitrogenase enzymes. Moreover, the structure-derived implications for CO_2_ capture by a surface-exposed [Fe_4_S_4_] cluster point to the possibility of developing novel strategies for CO_2_ sequestration while providing the initial insights into the unique mechanism of FeS-based CO_2_ activation.

## INTRODUCTION

Iron-sulfur (FeS) proteins utilize a wide array of FeS clusters to play key roles that range from electron transfer and catalysis to structural and regulatory functions in biological systems (1–7). A homodimer carrying a subunit-bridging [Fe_4_S_4_] cluster at the protein surface, the iron (Fe) protein of nitrogenase is best known for its function as an obligate electron donor for its catalytic partner during substrate turnover (8, 9). Recently, the Fe protein from a diazotrophic microbe, Azotobacter vinelandii (designated *Av*NifH) was shown to act as a reductase on its own and catalyze the ambient reduction of CO_2_ to CO via redox changes of its [Fe_4_S_4_] cluster (10). Interestingly, while the cluster of *Av*NifH is believed to cycle between the [Fe_4_S_4_]^1+^ (reduced) and [Fe_4_S_4_]^2+^ (oxidized) states (11–15) for its function as an electron donor in nitrogenase catalysis, catalytic turnover of CO_2_ by *Av*NifH on its own was observed when a strong reductant, europium(II) diethylenetriaminepentaacetic acid (Eu^II^-DTPA; *E*^0^′ = 1.14 V at pH 8.0), poised its cluster in the all-ferrous, [Fe_4_S_4_]^0^ state under *in vitro* conditions (10). Perhaps more interestingly, the Fe protein from a methanogenic microorganism, Methanosarcina acetivorans (designated *Ma*NifH), was capable of reducing CO_2_ past CO into hydrocarbons under ambient conditions in the presence of Eu^II^-DTPA, further illustrating the unique reactivity of the [Fe_4_S_4_] cluster toward CO_2_ (16, 17). Together, these observations point to the nitrogenase Fe protein as a simple model system for mechanistic investigations of FeS-based CO_2_ activation and reduction.

Of the two Fe protein species that have been investigated for their reactivity toward CO_2_, *Ma*NifH is particularly interesting given its ability to convert CO_2_ to CO and hydrocarbons. Despite its archaeal origin, *Ma*NifH shares a sequence identity of 59% and a sequence homology of 72% with *Av*NifH. Like *Av*NifH, *Ma*NifH is a homodimer of ∼60 kDa, and it contains an [Fe_4_S_4_] cluster that can adopt three oxidation states upon redox treatments: (i) the oxidized state ([Fe_4_S_4_]^2+^), which is generated upon treatment by indigodisulfonate; (ii) the reduced state ([Fe_4_S_4_]^1+^), which is generated upon treatment by dithionite (DT); and (iii) the “superreduced,” all-ferrous state ([Fe_4_S_4_]^0^), which is generated upon treatment by Eu^II^-DTPA (17). There are differences, however, in the electronic properties of *Ma*NifH and *Av*NifH, which are reflected by a stronger *S *=* *3/2 contribution to the electron paramagnetic resonance (EPR) spectrum of the reduced *Ma*NifH and a decreased intensity of the parallel mode, *g *=* *16.4 signal in the EPR spectrum of the superreduced *Ma*NifH (17). These differences, along with the lower reduction potential of the [Fe_4_S_4_]^1+/2+^ pair of *Ma*NifH (*E*^0^ = −395 mV) than that of *Av*NifH ([Fe_4_S_4_]^1+/2+^: *E*^0^ = −301 mV) (16), may contribute to the difference in the reactivities of *Ma*NifH and *Av*NifH toward CO_2_. The redox dependence of this reaction is further illustrated by a substantially decreased CO_2_-reducing activity of both *Ma*NifH and *Av*NifH in the presence of dithionite, a weaker reductant than Eu^II^-DTPA, which renders the clusters of these Fe proteins in the catalytically inefficient [Fe_4_S_4_]^1+^ state (10, 16).

The significantly decreased activity of Fe protein in a dithionite-driven reaction could prove advantageous for capturing CO_2_ in an early stage of CO_2_ reduction. Here, we report a 2.4-Å crystal structure of *Ma*NifH that was generated in the presence of dithionite and an alternative CO_2_ source, bicarbonate. Structural analysis of this previously uncharacterized Fe protein from the methanogen nitrogenase family suggests that CO_2_ is possibly captured in an unactivated, linear conformation on the dithionite-reduced *Ma*NifH; moreover, it reveals the initial coordination of CO_2_ by a conserved, surface-exposed arginine (Arg) pair in a concerted yet asymmetric manner, which could assist in trapping CO_2_ near the [Fe_4_S_4_] cluster via hydrogen bonding and electrostatic interactions. These results provide a useful framework for further exploration of the mechanism of CO_2_ activation by Fe proteins, which may enable future development of FeS catalysts for recycling the greenhouse gas CO_2_ into valuable chemical commodities.

## RESULTS

### Structural analysis of the dithionite-reduced *Ma*NifH.

**FIG 1:**
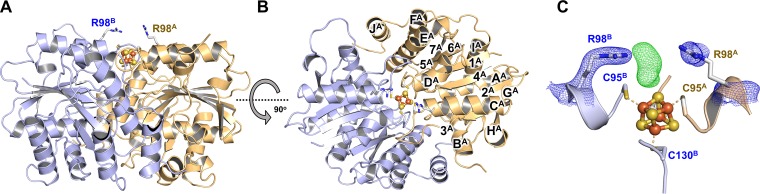
Side (A) and top (B) views of the 2.4-Å crystal structure of *M*aNifH. The subunits are shown as ribbons (subunit A, light orange; subunit B, light blue). The α-helices (A^A^-J^A^) and β-sheets (1^A^-7^A^) of subunit A are indicated. The [Fe_4_S_4_] cluster is shown in ball-and-stick presentation (Fe, orange; S, yellow). (C) The electron density (2*F*_o_ − *F*_c_) of the active site of *Ma*NifH was contoured at 1.5-σ level for the conserved Arg pair (blue meshes), and the omit map (*F*_o_ − *F*_c_) of the additional electron density (green mesh) that is unaccounted for in the structure was contoured at 3.0-σ level. The four Cys ligands (C95^A^, C130^A^, C95^B^, C130^B^) and the conserved Arg pair (R98^A^, R98^B^) are shown as sticks.

Consistent with the presence of its [Fe_4_S_4_] cluster in the +1 oxidation state, *Ma*NifH crystallized in the presence of dithionite had a characteristic brown color. The ∼2.4-Å structure of the dithionite-reduced *Ma*NifH (PDB ID 6NZJ) adopts the same overall conformation as all Fe protein structures reported to date (9, 18–20), with each of its subunits folded as a single α/β-type domain and its [Fe_4_S_4_] cluster situated in a surface cavity between the two subunits (Fig. 1A and B). A closer examination of the region surrounding the active site of *Ma*NifH (Fig. 1C) reveals the ligation of the [Fe_4_S_4_] cluster by four Cys residues: two from subunit A (Cys95^A^, Cys130^A^) and two from subunit B (Cys95^B^, Cys130^B^). Interestingly, the electron density omit map (*F*_o_ − *F*_c_) of the active site of *Ma*NifH (Fig. 1C, green mesh; also see Fig. S1 in the supplemental material) indicates the presence of additional electron density that lies immediately next to the crystallographic symmetry axis, seemingly held by two pairs of conserved Arg residues (R98^A^ and R98^B^)—one from each of the two adjacent *Ma*NifH subunit dimers.

10.1128/mBio.01497-19.1 FIG S1 Structure of *Ma*NifH without additional ligand. The subunits are shown as ribbons and colored light orange (subunit A) and light blue (subunit B) for the asymmetric unit and yellow (subunit A) and dark blue (subunit B) for the symmetry mate. The *F*_o_ – *F*_c_ map (green mesh) is contoured at 3 σ. The side chains of R98 are shown as sticks. The [Fe_4_S_4_] clusters are shown in ball-and-stick presentation and colored as follows: Fe, orange; S, yellow. Download FIG S1, JPG file, 0.8 MB. Copyright © 2019 Rettberg et al. 2019 Rettberg et al. This content is distributed under the terms of the Creative Commons Attribution 4.0 International license.

### Modeling the extra electron density in the structure of the dithionite-reduced *Ma*NifH.

Given that the additional electron density may originate from the small molecules in the protein preparations or crystallographic solutions, we then considered possible candidates and modeled water (see Fig. S2A in the supplemental material), carbonate (Fig. S2B), glycerol (Fig. S2C), and CO_2_ (Fig. S2D), respectively, into this density. Water is an unlikely contributor to this density, as modeling of one water molecule in the asymmetric unit and another in its symmetry mate results in substantial “leftover” electron density in the *F*_o_ − *F*_c_ omit map (Fig. S2A, green mesh). Carbonate and glycerol, on the other hand, could be modeled as two molecules—each at ∼50% occupancy—at the crystallographic symmetr*y* axis with reasonable *R* factor values (see Table S1 in the supplemental material). Similarly, CO_2_ could be modeled with reasonable *R* factor values at the crystallographic symmetry axis; only in this case, two molecules of CO_2_—each at 100% occupancy—could be assigned to the asymmetric unit and its symmetry mate, respectively (Table S1). It should be noted that the modeling of two CO_2_ moieties results in some negative electron density; however, the overall crystallographic statistics are reasonable to support this model (Table S1) despite the difficulty to conclusively assign this ligand near the crystallographic symmetr*y* axis.

10.1128/mBio.01497-19.2 FIG S2 Structures of *Ma*NifH modeled with two water molecules (A), two carbonate molecules (B), two glycerol molecules (C), and two CO_2_ moieties (D), respectively. The subunits are shown as ribbons and colored light orange (subunit A) and light blue (subunit B) for the asymmetric unit and yellow (subunit A) and dark blue (subunit B) for the symmetry mate. The *F*_o_ – *F*_c_ map (red mesh) is contoured at −3 σ, the *F*_o_ – *F*_c_ map (green mesh) is contoured at 3 σ, and the 2*F*_o_ – *F*_c_ map (blue mesh) is contoured at 1.5 σ. One water (A) or CO_2_ (B) molecule was modeled in the asymmetric unit and the other in the symmetry mate, respectively. The two carbonate (B) or glycerol (C) molecules were placed on the crystallographic axis. The side chains of R98 are shown as sticks. The [Fe_4_S_4_] cluster and the water, carbonate, and glycerol moieties are shown in ball-and-stick presentation and colored as follows: Fe, orange; S, yellow; C, gray; O, red. Download FIG S2, JPG file, 2.3 MB. Copyright © 2019 Rettberg et al. 2019 Rettberg et al. This content is distributed under the terms of the Creative Commons Attribution 4.0 International license.

10.1128/mBio.01497-19.7 TABLE S1 Data collection, refinement, and ligand fitting statistics of *Ma*NifH (PDB ID 6NZJ). Shown are the values for the ligand-free model (upper) and the statistics for the models with the plausible ligands CO_2_, glycerol, and carbonate (lower). Download Table S1, PDF file, 0.1 MB. Copyright © 2019 Rettberg et al. 2019 Rettberg et al. This content is distributed under the terms of the Creative Commons Attribution 4.0 International license.

### DFT calculations of the affinity of CO_2_ to the dithionite-reduced *Ma*NifH.

**FIG 2:**
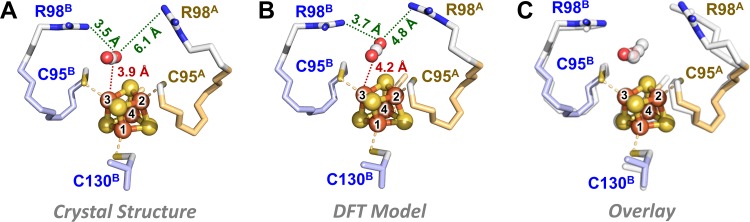
Crystal (A) and DFT-optimized (B) structures of *Ma*NifH with the extra electron density modeled as CO_2_ and (C) an overlay of the two structures. The conserved pair of Arg residues assume “proximal” (R98^B^) and “distal” (R98^A^) positions, respectively, to the CO_2_ moiety and the Fe-3 atom of the cluster (A and B), and CO_2_ occupies a highly similar position in the crystal structure and the DFT model (C). The [Fe_4_S_4_] cluster and CO_2_ moiety are shown in ball-and-stick presentation and colored as follows: Fe, orange; S, yellow; C, gray; O, red. The Cys ligands and the conserved Arg residues are shown as sticks.

To seek support for the assignment of CO_2_ as the extra electron density in the crystal structure of *Ma*NifH, we then used density functional theory (DFT) calculations to analyze the CO_2_ affinity of the [Fe_4_S_4_]^1+^ cluster in *Ma*NifH. Consistent with our previous findings for both *Av*NifH-bound and synthetic [Fe_4_S_4_] clusters (10, 16), CO_2_ does not interact well with the [Fe_4_S_4_]^1+^ cluster of *Ma*NifH and tends to dissociate from the cluster during the course of structural optimization; however, the two highly conserved Arg residues in *Ma*NifH (R98^A^, R98^B^) form a cage-like configuration around the CO_2_ molecule that assists in trapping it in close proximity to the cluster (see Movie S1 in the supplemental material). Interestingly, the location of the CO_2_ moiety in the DFT-optimized model is in good agreement with half of the electron density pattern in the structure of *Ma*NifH, except for a slight reorientation of CO_2_ (Fig. 2). In comparison, DFT optimization reveals protonation of carbonate by R98^B^, followed by coordination of the resulting bicarbonate in a position parallel to the upper surface of the [Fe_4_S_4_] cluster, which is rather distinct from the perpendicular position modeled for carbonate in the crystal structure of *Ma*NifH (see Fig. S3 in the supplemental material). This observation is important, as it provides theoretical support for the assignment of CO_2_ as a potential ligand in the structure of the dithionite-reduced *Ma*NifH protein. The fact that the *Ma*NifH crystals were generated at a bicarbonate concentration in the same order of magnitude as that used to generate a CO_2_-bound conformation of CO dehydrogenase (21) provides further support for the assignment of CO_2_ in the *Ma*NifH structure. In this scenario, the CO_2_ moiety has its C atom placed at a distance of ∼4 Å from the nearest Fe atom (Fe-3) of the [Fe_4_S_4_] cluster, with the NH_2_^+^ groups of R98^A^ and R98^B^ assuming the “distal” and “proximal” positions, respectively, to Fe-3 (Fig. 2). This observation suggests a possible role of the conserved Arg pair in capturing CO_2_ via hydrogen bonding and/or electrostatic interactions, as well as a potentially asymmetric functionality of the two Arg residues in this process.

10.1128/mBio.01497-19.3 FIG S3 Crystal structure of *Ma*NifH with the extra electron density modeled as carbonate (A) and DFT-optimized structure of *Ma*NifH with carbonate (B) or bicarbonate (C) as the substrate. In the crystal structure, the carbonate moiety assumes a position perpendicular to the upper surface of the [Fe_4_S_4_] cluster (A). Based on DFT-based structural optimization, however, carbonate is first protonated by R98^B^, and the resulting bicarbonate assumes a position parallel to the upper surface of the [Fe_4_S_4_] cluster (B). The DFT-optimized structure calculated directly with bicarbonate (C) closely resembles that achieved with carbonate upon protonation (B). Overall, carbonate occupies a different position in the crystal structure than (bi)carbonate in the DFT models, which can be best visualized by the overlay of the carbonate-bound crystal structure with the (bi)carbonate-bound DFT models that originate either from the protonation of carbonate (D) or directly from bicarbonate (E). In both cases, the (bi)carbonate moiety swings ∼90° from a position that is perpendicular to one that is parallel to the upper surface of the [Fe_4_S_4_] cluster. Furthermore, the (bi)carbonate moiety rotates ∼90° counterclockwise in a horizontal plane parallel to the upper surface of the cluster. The hydrogen atoms of bicarbonate are omitted from the figure, as they cannot be discerned by X-ray crystallography. The [Fe_4_S_4_] cluster and carbonate/bicarbonate moieties are shown in ball-and-stick presentations and colored as follows: Fe, orange; S, yellow; C, gray; O, red. The Cys ligands and the conserved Arg residues are shown as sticks. Download FIG S3, JPG file, 1.4 MB. Copyright © 2019 Rettberg et al. 2019 Rettberg et al. This content is distributed under the terms of the Creative Commons Attribution 4.0 International license.

10.1128/mBio.01497-19.8 MOVIE S1 Course of DFT-based structural optimization (TPSS/def2-SVP/def2-TZVP) of the CO_2_-captured conformation of *Ma*NifH in the [Fe_4_S_4_]^1+^ state (*S *=* *1/2). The CO_2_ moiety in the DFT-optimized structure is rendered gray, and the CO_2_ moiety in the crystal structure is colored as follows: C, gray; O, red. Download Movie S1, MPG file, 7.8 MB. Copyright © 2019 Rettberg et al. 2019 Rettberg et al. This content is distributed under the terms of the Creative Commons Attribution 4.0 International license.

### Examining the role of the conserved Arg pair of *Ma*NifH in CO_2_ capture.

**FIG 3:**
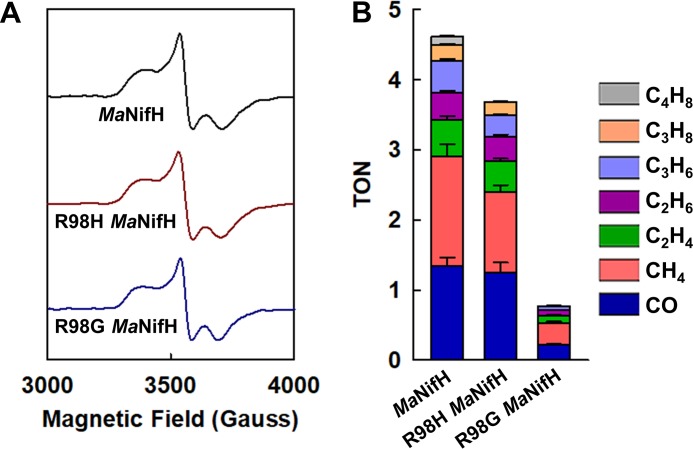
Spectroscopic and catalytic features of the wild-type and variant *Ma*NifH. (A) EPR spectra and (B) CO_2_-reducing activities of wild-type and variant *Ma*NifH. EPR spectra were collected at 10 K. The wild-type and R98H and R98G variant *Ma*NifH are dimers of ∼60 kDa and contain 3.7 ± 0.1, 3.9 ± 0.4, and 3.8 ± 0.2 nmol Fe per nmol protein, respectively. Like the wild-type *Ma*NifH, the R98H and R98G variants display the same [Fe_4_S_4_]^+^ characteristic, *S *=* *1/2 EPR signal in the dithionite-reduced state (A), yet they display disparate activities in CO_2_ reduction (B). The hydrocarbon/CO ratios (calculated based on total nmol of reduced carbons) of the wild-type *Ma*NifH and R98H variant are 2.7 and 1.9, respectively, suggesting a shift from hydrocarbon formation to CO formation in the latter case.

To test the proposed role of conserved Arg residues in CO_2_ capture, we performed site-directed mutagenic analysis and mutated R98 of *Ma*NifH to either a His or a Gly. Both R98H and R98G *Ma*NifH variants display the same *S *=* *1/2 EPR signal as the wild-type protein, which is indicative of an unperturbed [Fe_4_S_4_] center in the +1 oxidation state (Fig. 3A). However, the R98H variant of *Ma*NifH retains ∼80% CO_2_-reducing activity, whereas the R98G variant loses ∼85% of this activity (Fig. 3B), consistent with the preservation (i.e., the R→H mutation) or elimination (i.e., the R→G mutation) of the hydrogen bonding ability at the position of R98. The somewhat decreased activity of the R98H variant could be explained by a shorter side chain of His and, consequently, a reduced efficiency of this residue in hydrogen bonding/proton donation than Arg. The slight defect of His in proton donation would also account for a shift of the product profile of the R98H variant (hydrocarbon/CO ratio of 1.9) from hydrocarbon formation to CO formation compared to that of the wild-type *Ma*NifH (hydrocarbon/CO ratio of 2.7), as formation of CO requires fewer protons than that of hydrocarbons.

### Proposal of a plausible mechanism for the initial capture of CO_2_ by *Ma*NifH.

**FIG 4:**
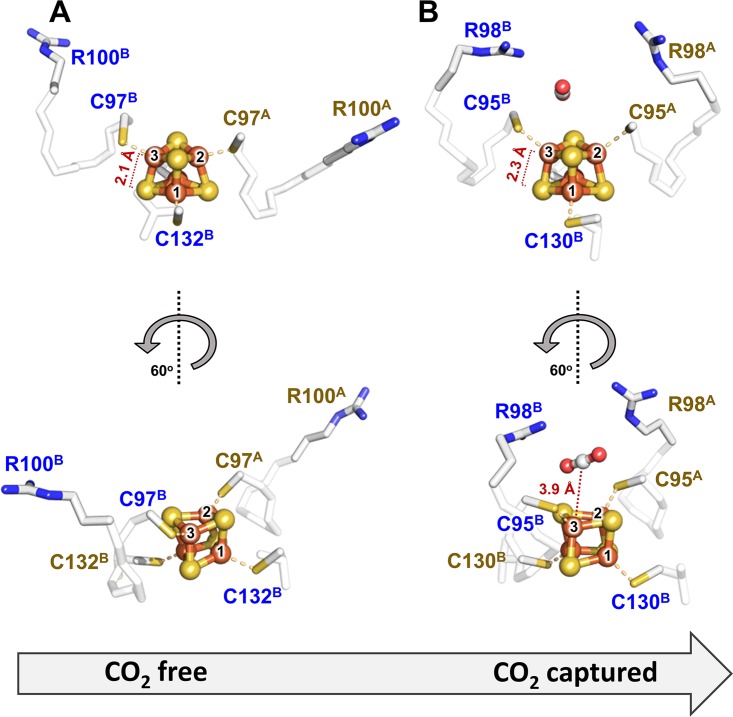
Comparison of the CO_2_-free (A) and CO_2_-captured (B) conformations of Fe protein, showing concerted yet asymmetric movement of a pair of conserved Arg residues that potentially capture CO_2_ near the [Fe_4_S_4_] cluster. The CO_2_-free and CO_2_-captured conformations are represented by the homologous *Av*NifH and *Ma*NifH, respectively. The movement of the “proximal” Arg (R100^B^ in *Av*NifH and the corresponding R98^B^ in *Ma*NifH) and the “distal” Arg (R100^A^ in *Av*NifH and the corresponding R98^A^ in *Ma*NifH) is shown from two angles.

To obtain further insights into the mechanism of CO_2_ capture by nitrogenase Fe proteins, we compared our dithionite-reduced *Ma*NifH structure (PDB ID 6NZJ) that is potentially bound with CO_2_ with a previously reported, dithionite-reduced *Av*NifH structure (PDB ID 1G5P) that is free of CO_2_ (19). Consistent with a high degree of sequence homology between *Ma*NifH and *Av*NifH, the subunits A and B in *Ma*NifH show Cα deviations of only 0.599 and 0.616 Å, respectively, relative to those in *Av*NifH, yet the two subunit chains in *Ma*NifH are more similar to each other in terms of secondary structural elements, particularly with respect to the structurally less conserved α-helical regions (see Fig. S4 and S5 in the supplemental material). More strikingly, compared to their counterparts in *Av*NifH, there is a notable movement of the two subunits of *Ma*NifH with respect to each other, which flattens the surface cavity and consequently “pushes” the [Fe_4_S_4_] cluster further toward the surface where a CO_2_ molecule could be modeled (see Fig. S6 and Movie S2 in the supplemental material). A top-view comparison between the two structures further reveals a “linearization” of helices C^A^ and C^B^ in *Ma*NifH relative to those in *Av*NifH, which is accompanied by a substantial swing of the Arg pair, R98^A^ and R98^B^ (located at the tips of helices C^A^ and C^B^), toward the center of the surface cavity (see Fig. S6 and Movie S3 in the supplemental material). Such a movement of the conserved Arg pair could reflect a concerted action of the “distal” R98^A^ and the “proximal” R98^B^ in the initial capture of CO_2_ in an unactivated, linear conformation near the Fe-3 atom of the [Fe_4_S_4_] cluster (Fig. 4). Further activation of CO_2_ into a bent, carboxylate-like conformation may continue to employ an asymmetric mechanism. Previous DFT calculations of CO_2_ activation by the catalytically competent, all-ferrous *Av*NifH (10) led to the proposal of binding of an activated CO_2_ moiety via coordination of C with Fe-3 of the cluster and coordination of O with the guanidinium group of the “proximal” R100^B^ (corresponding to the “proximal” R98^B^ in *Ma*NifH), with the latter potentially donating protons for the subsequent C-O bond cleavage.

10.1128/mBio.01497-19.4 FIG S4 Identities between subunits A and B within *Av*NifH and *Ma*NifH. (A and B) Percentages of identities between individual α-helices (A) and β-sheets (B) of subunits A and B within *Av*NifH (black) and *Ma*NifH (red). (C and D) Overall percentages of identities between α-helices (C) and β-sheets (D) of subunits A and B within *Av*NifH (black) and *Ma*NifH (red). Data from Fig. S5 were used to calculate these percentages. Cα deviations of subunit A versus subunit B: *Av*NifH, 0.356 Å, and *Ma*NifH, 0.334 Å. Download FIG S4, JPG file, 0.8 MB. Copyright © 2019 Rettberg et al. 2019 Rettberg et al. This content is distributed under the terms of the Creative Commons Attribution 4.0 International license.

10.1128/mBio.01497-19.5 FIG S5 Subunits A and B of *Av*NifH and *Ma*NifH. Shown are comparisons between (A and B) primary sequences and (C and D) secondary structures of subunits A (A and C) and B (B and D) of *Av*NifH and *Ma*NifH. The identical residues in the primary sequences are indicated with *, and the conserved Cys ligands for the [Fe_4_S_4_] cluster are shown in red (A and B). The α-helices are colored green, the β-sheets are colored cyan (C and D), and the primary sequences corresponding to these structural elements are highlighted with the corresponding colors (A and B). Download FIG S5, JPG file, 2.8 MB. Copyright © 2019 Rettberg et al. 2019 Rettberg et al. This content is distributed under the terms of the Creative Commons Attribution 4.0 International license.

10.1128/mBio.01497-19.6 FIG S6 Structural comparison between *Av*NifH and *Ma*NifH. Shown are side (left) and top (middle) views of *Av*NifH (A) and *Ma*NifH (B), with the alignments of helices C^B^ and C^A^ and the positions of the Arg pairs (located at the tips of helices C^B^ and C^A^) highlighted (right). The movements of Arg residues in *Ma*NifH (B, right) relative to those in *Av*NifH are indicated by dashed red arrows. Subunits are shown as ribbons (side view) or cylinders (top view) and colored light orange (subunit A) and light blue (subunit B), respectively. The [Fe_4_S_4_] cluster and CO_2_ moiety are shown in ball-and-stick presentation and colored as follows: Fe, orange; S, yellow; C, gray; O, red. Download FIG S6, JPG file, 2.3 MB. Copyright © 2019 Rettberg et al. 2019 Rettberg et al. This content is distributed under the terms of the Creative Commons Attribution 4.0 International license.

10.1128/mBio.01497-19.9 MOVIE S2 Conformational morphing of *Av*NifH into *Ma*NifH (side view). Both structures are depicted and colored as described in the legend to Fig. S1. The conformational morphing plugin of PyMol 2.1.1 (https://pymol.org) was used to generate the movie, with *Av*NifH as the start conformation and *Ma*NifH as the end conformation. Refinement cycles, 3; number of output states, 100; interpolation method, RigiMOL. Download Movie S2, MPG file, 7.9 MB. Copyright © 2019 Rettberg et al. 2019 Rettberg et al. This content is distributed under the terms of the Creative Commons Attribution 4.0 International license.

10.1128/mBio.01497-19.10 MOVIE S3 Conformational morphing of *Av*NifH into *Ma*NifH (top view). The movie was generated as described in the legend to Movie S2. Download Movie S3, MPG file, 6.6 MB. Copyright © 2019 Rettberg et al. 2019 Rettberg et al. This content is distributed under the terms of the Creative Commons Attribution 4.0 International license.

## DISCUSSION

In light of a plausible asymmetric mechanism of CO_2_ activation by Fe protein, it is interesting to consider the mechanism proposed for the Ni-dependent CO dehydrogenase in CO_2_ activation, which involves the action of the Fe/Ni atoms of its heterometallic C-cluster ([NiFe_4_S_4_]) as a pair of Lewis acid/base to facilitate scission of a C-O bond (21–24). In the absence of such a heterometal-based asymmetry, it is plausible that activation of CO_2_ by the homometallic [Fe_4_S_4_] cluster would resort to a structure-based asymmetry that enables interactions between O and the guanidinium group of the proximal Arg, as well as binding of C to the nearest Fe-3 atom. It is worth noting that the proposed asymmetric functionality of the conserved Arg pair in CO_2_ activation is consistent with the previously established regulatory mechanism of nitrogenase activity through ADP-ribosylation of only one of these conserved Arg residues (25), whereas the structure-based suggestion of a single reactive Fe (Fe-3) site for CO_2_ activation may have certain relevance to the unique Fe site that was identified by earlier Mössbauer studies of the all-ferrous Fe protein (14). While the functions of these asymmetric elements await further elucidation, the current study provides a useful framework for investigating the structural basis of Fe protein-based CO_2_ capture and activation. Moreover, the strategy utilized by the Fe protein to trap CO_2_ by a pair of surface-located arginines loosely resembles the approaches that employ nitrogen-based ligands, such as metal-organic frameworks (MOFs) with amine or amide groups (26) or protein amyloid fibers comprising lysines in stacked sheets (27), for CO_2_ capture and sequestration. The fact that the arginine residues of the Fe protein trap CO_2_ in the close proximity to a surface-exposed [Fe_4_S_4_] cluster for further processing may provide a conceptual basis for the future development of MOF- or protein-based FeS catalysts that couple the capture of CO_2_ with the recycling of this greenhouse gas into useful chemical commodities.

## MATERIALS AND METHODS

### Protein purification and crystallization.

All protein purification steps were carried out anaerobically using Schlenk techniques. His-tagged *Ma*NifH was purified by immobilized metal affinity as described elsewhere (17, 28). Reagents for protein crystallization were purchased from Hampton Research and were thoroughly deaerated by vacuum/Ar-fill cycling before use. All crystals were generated at room temperature in an anaerobic chamber (Coy Laboratory Products), coated with Parabar 10312 oil (Hampton Research) as a cryo-protectant, and flash-frozen in liquid nitrogen for data collection.

*Ma*NifH was crystallized at room temperature by a microbatch method under a layer of Al’s oil (Hampton Research). The purified *Ma*NifH protein was desalted on a G-25 fine column equilibrated with buffer M (10 mM EPPS [pH 8.0], 100 mM NaCl, 10% [vol/vol] glycerol, and 2 mM dithionite [DT]) and then concentrated to 10 mg/ml by Amicon Ultra-4 30-kDa centrifugal filter units. The crystals were grown by evaporating a mixture of 1 μl protein solution and 3 μl precipitant solution (2.3 M ammonium sulfate, 7% [wt/vol] polyethylene glycol 3350 [PEG 3350], 12 mM carbonate, and 2 mM DT) under Al’s oil. The protein solution was brown, indicating that the protein-bound cluster was present in the reduced, +1 state. Brown crystals grew after 2 weeks and were flash-frozen in liquid nitrogen for data collection.

### Data collection and structural determination.

The diffraction data of *Ma*NifH crystals were collected at 100 K on beamline 8.2.1 of Advanced Light Source using a wavelength of 0.9774 Å and an ADSC Q315r charge-coupled device (CCD) detector. A total of 501 images were recorded for *Ma*NifH at a distance of 450 mm, with an oscillation angle of 0.25° and an exposure time of 0.25 s. The raw data were indexed and processed using iMosflm and Scala in the CCP4 package (29). Molecular replacement was performed with Phaser in PHENIX (30) using the structure of the Clostridium pasteurianum NifH protein (PDB ID 1CP2) (19) as a search model. The initial model was further improved by cycles of manual building and refinement using Coot and PHENIX (30–32). At the end of the refinement cycle, water, carbonate, glycerol, or CO_2_ was manually put into the model of *Ma*NifH and further refined for 3 cycles using PHENIX. The stereochemical quality of the final structures was evaluated by MolProbity (33). All molecular graphics were prepared using PyMol (34). Data collection and statistics for refinement and ligand modeling are summarized in Table S1.

### Strain construction and activity analyses.

Strains expressing R98H and R98G *Ma*NifH variants were constructed via site-directed mutagenesis of the wild-type Methanosarcina acetivorans *nifH* sequence carried on a pET14b vector (17), followed by transformation of the resultant plasmids into Escherichia coli strain BL21(DE3). The *in vitro* CO_2_-reduction assays were carried out in 9.4-ml assay vials with crimped butyl rubber serum stoppers. Each assay contained, in a total volume of 1.0 ml, 500 mM Tris-HCl (pH 10.0), 0.5 mg Fe protein (wild-type or R98H or R98G variant *Ma*NifH), and 100 mM Eu^II^-DTPA. In addition, the headspace of each assay contained 100% CO_2_ (for reactions) or 100% Ar (for controls). The assays were assembled without protein and Eu^II^-DTPA and repeatedly flushed and exchanged with CO_2_, followed by equilibration for 30 min until pH stabilized at ∼8.0. The reaction was initiated upon addition of *Ma*NifH, followed immediately by addition of Eu^II^-DTPA and incubation with continuous shaking at 30°C for 300 min until the reaction was complete. Following the quenching of each assay by 100 μl of 30% trichloroacetic acid, the headspace sample was examined for the production of CO and hydrocarbons as described previously (16).

### EPR spectroscopy analyses.

The EPR samples were prepared in a Vacuum Atmospheres glove box and flash-frozen in liquid nitrogen prior to analysis. The DT-reduced samples contained 2 mM DT, 50 mM Tris-HCl (pH 8.0), 500 mM NaCl, and 10% (vol/vol) glycerol. EPR spectra were recorded by an ESP 300 Ez spectrophotometer (Bruker) interfaced with an ESR-9002 liquid-helium continuous-flow cryostat (Oxford Instruments) using a microwave power of 50 mW, a gain of 5 × 10^4^, a modulation frequency of 100 kHz, and a modulation amplitude of 5 G. Five scans were recorded for each EPR sample at a temperature of 10 K and a microwave frequency of 9.62 GHz.

### Density functional theory calculations.

The mechanism of CO_2_, carbonate, and bicarbonate coordination was studied with the DFT programs in the Turbomole package, version 7.0 (35). Atomistic models of the [Fe_4_S_4_] cluster and its immediate protein environment were generated from the structure of *Ma*NifH (PDB ID 6NZJ [this work]) in the DT-reduced, [Fe_4_S_4_]^1+^ state.

The models were selected as described previously (10) and contained the [Fe_4_S_4_] cluster and C95^A^, C95^B^, C130^A^, C130^B^, R98^A^, R98^B^, F133^A^, F133^B^, and the main-chain atoms of the residues A96^A^, A96^B^, A97^A^, G97^B^, G131^A^, G131^B^, G132^A^, and G132^B^ of *Ma*NifH to account for all interactions of the cluster with the protein backbone. N termini were saturated with acetyl groups according to the crystallographic atom positions. Hydrogen atoms were added to the model with Open Babel (36), assuming protonation of the Arg residues. During structural optimizations, the atoms of the cluster, the side-chain atoms of the cluster-coordinating Cys residues (including Cα), the side-chain atoms of the Arg residues (starting from Cγ), the benzene groups of the Phe residues, and all hydrogen atoms were allowed to spatially relax. All other atoms were kept structurally frozen. The models were treated as open-shell systems in the unrestricted Kohn-Sham framework. Solvent effects were treated implicitly by the conductor-like solvent screening model (COSMO) (37), assuming a dielectric constant of ɛ = 40. The structures were optimized with the TPSS (Tao-Perdew-Staroverov-Scuseria) functional (38). A def2-TZVP basis set (39, 40) was used for the [Fe_4_S_4_] cluster, the side-chain atoms of the Cys residues (including Cα atoms), the atoms of the guanidinium groups, and the cluster-bound CO_2_, carbonate, and bicarbonate moieties. A def2-SVP basis set was assigned to all remaining atoms to accelerate the calculations. Computational time was further reduced by utilizing the resolution-of-the-identity approximation (41, 42). Antiferromagnetic coupling in the FeS cluster was accounted for by the broken symmetry approach (43–45).

### Data availability.

The structure of DT-reduced *Ma*NifH (PDB ID 6NZJ) has been deposited in the Protein Data Bank (https://www.wwpdb.org) and will be released upon publication.
