# Supplementary figures and images for: Structural Analysis of a Nitrogenase Iron Protein from Methanosarcina acetivorans: Implications for CO2 Capture by a Surface-Exposed [Fe4S4] Cluster
Source: mBio. 2019 Jul 9;10(4):e01497-19. doi: 10.1128/mBio.01497-19 (PMC6747716; doi:10.1128/mBio.01497-19)

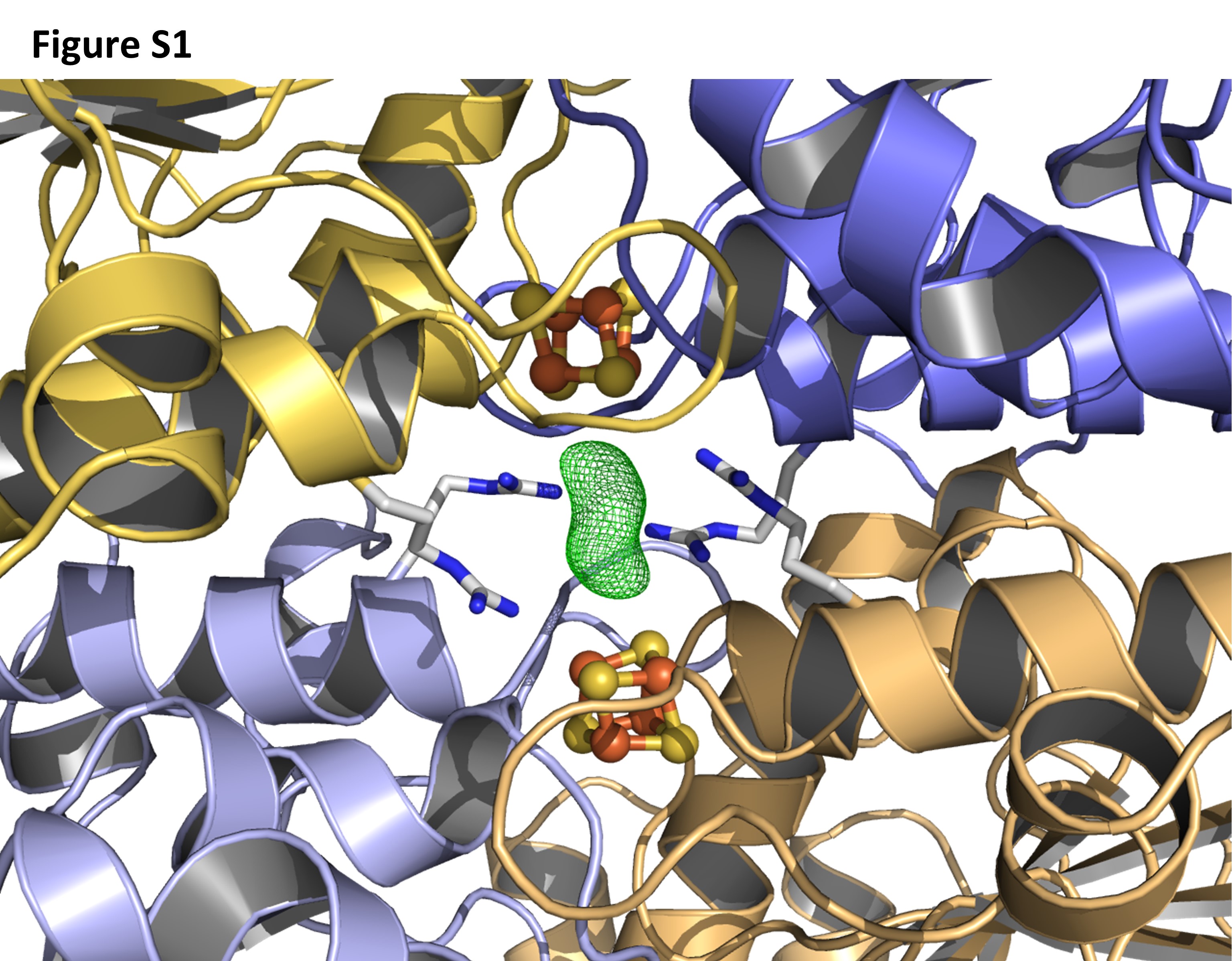

Supplement: FIG S1 [file mBio.01497-19-sf001.jpg]

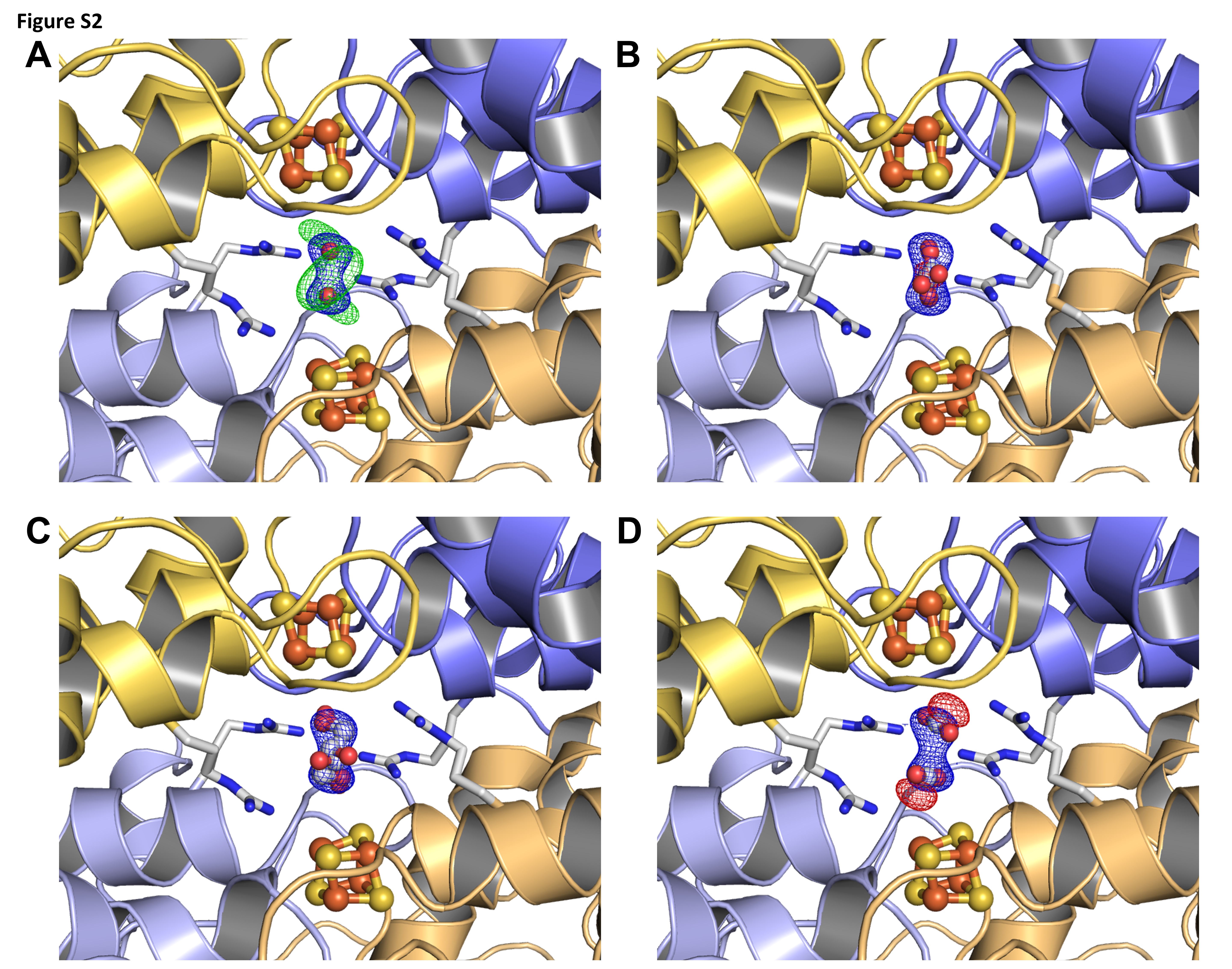

Supplement: FIG S2 [file mBio.01497-19-sf002.jpg]

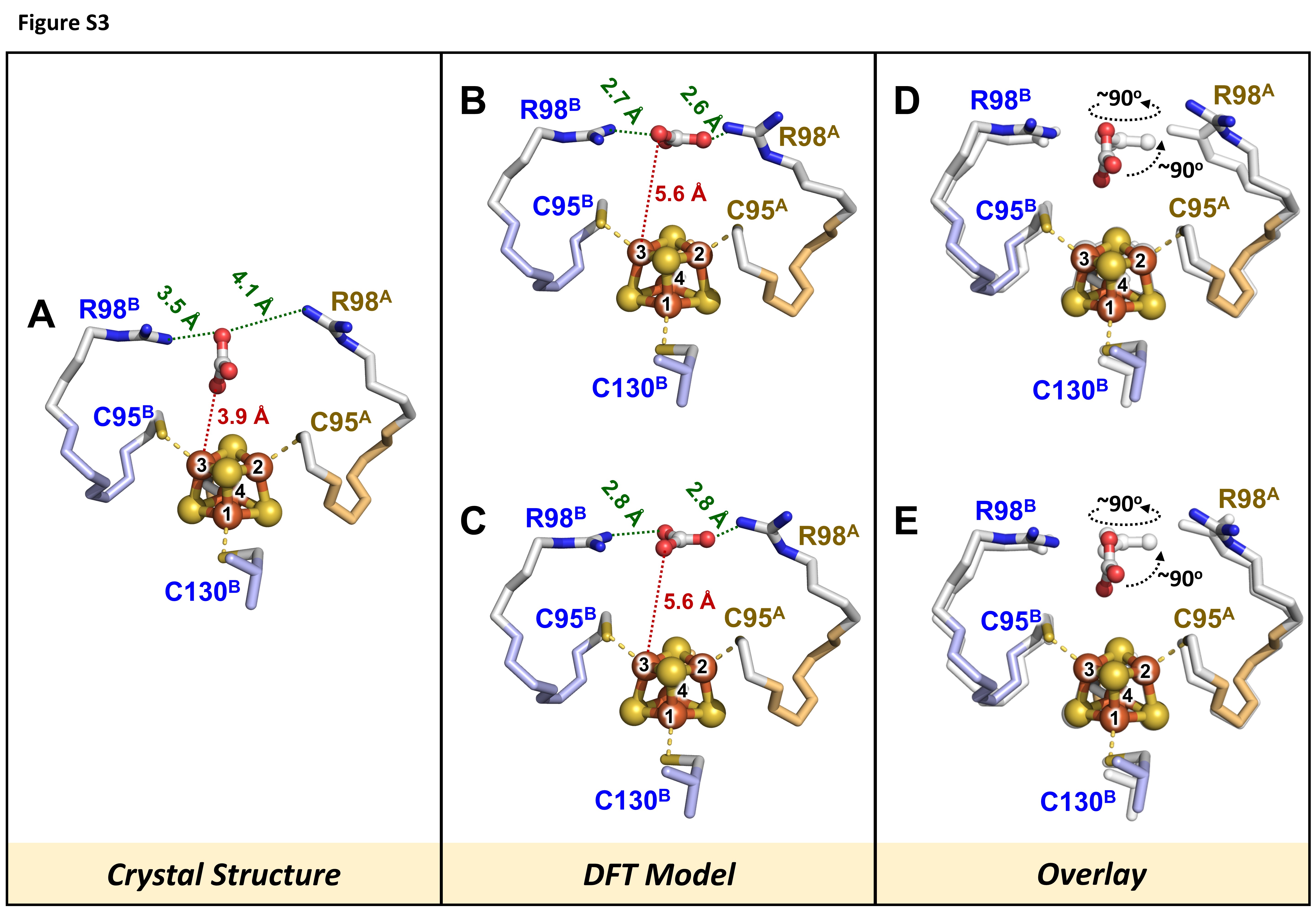

Supplement: FIG S3 [file mBio.01497-19-sf003.jpg]

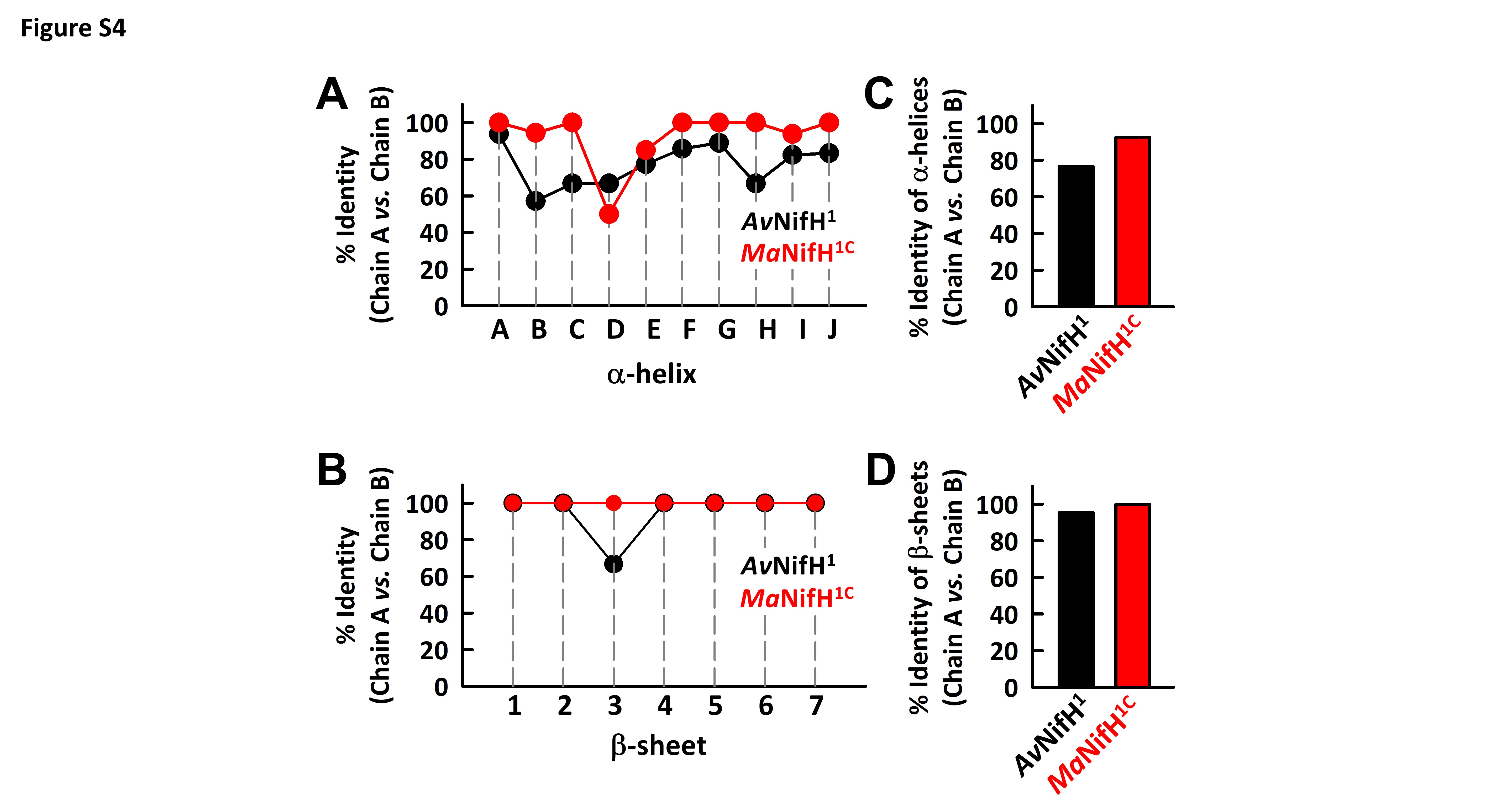

Supplement: FIG S4 [file mBio.01497-19-sf004.jpg]

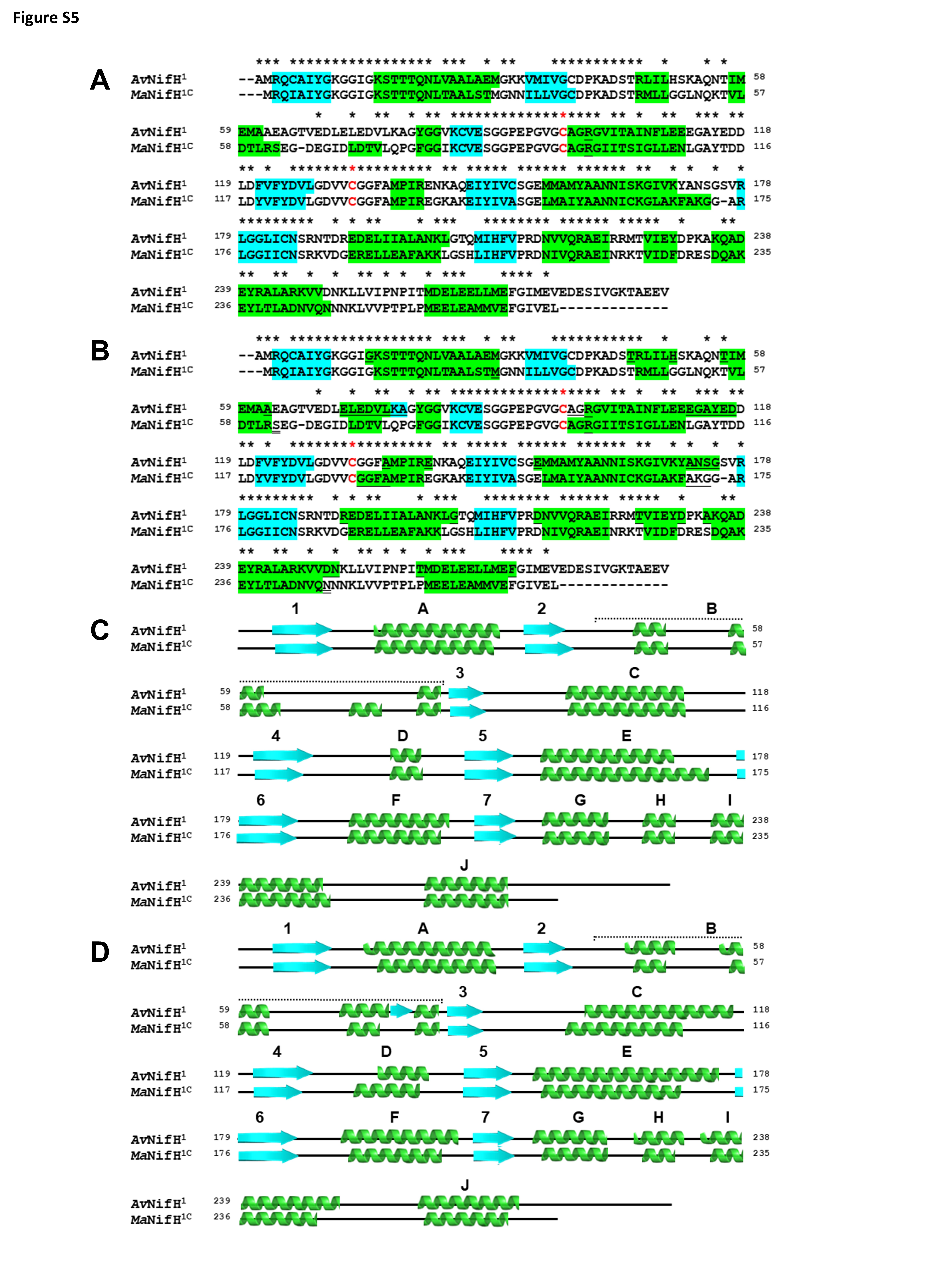

Supplement: FIG S5 [file mBio.01497-19-sf005.jpg]

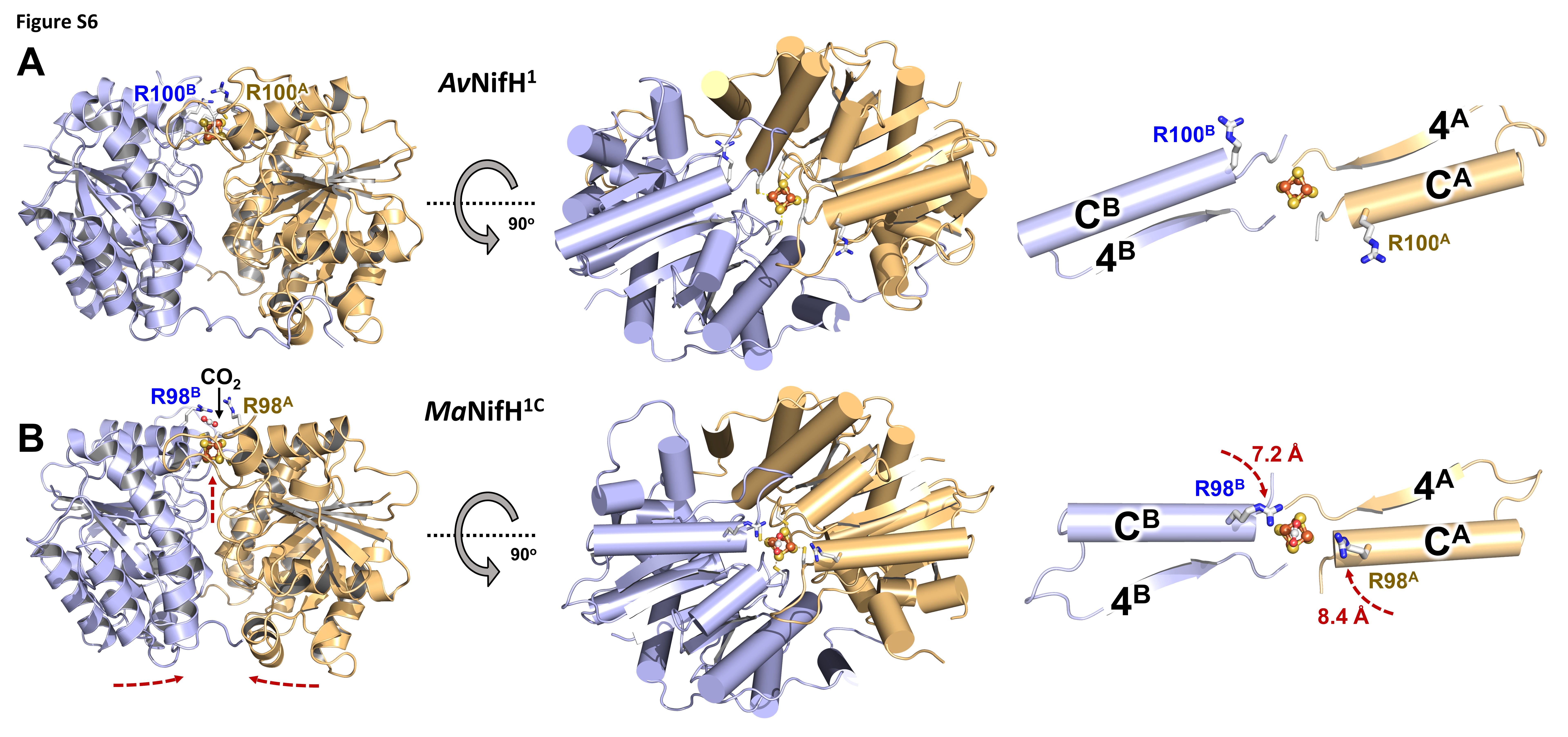

Supplement: FIG S6 [file mBio.01497-19-sf006.jpg]
