# Supplementary material for: Structural Analysis of a Nitrogenase Iron Protein from Methanosarcina acetivorans: Implications for CO2 Capture by a Surface-Exposed [Fe4S4] Cluster
Source: mBio. 2019 Jul 9;10(4):e01497-19. doi: 10.1128/mBio.01497-19 (PMC6747716; doi:10.1128/mBio.01497-19)
Supplement: TABLE S1 [file mBio.01497-19-st001.pdf]

Table S1. Data collection, refinement and ligand model statistics of *Ma*NifH (PDB ID 6NZJ)

Space group	$\boldsymbol{P6_522}$
Cell dimensions	
a, b, c (Å)	96.16, 96.16, 320.24
α, β, γ (°)	90.00, 90.00, 120.00
Wavelength (Å)	0.97741
Number of reflections measured	419,306 (35,917)
Number of unique reflections	35,959 (3,432)
Resolution (Å)	83.3 – 2.4 (2.5-2.4)[a]
R_{meas} (%)	11.1 (78.1)
$CC_{1/2}$	0.999 (0.810)
Mean I/σ(I)	5.4 (1.0)
Completeness (%)	100.0 (99.9)
Multiplicity	11.9 (11.2)
Resolution (Å)	83.3 – 2.4
No. of reflections	35,369
R_{work} / R_{free} [b] (%)	18.31 / 21.80
Number of atoms (non-H)	
Overall	4,147
Protein	3,952
Ligand	23
Solvent	172
Average B factors (Å^2)	
Overall	49.01
Protein	48.95
Ligand	42.46
Water	51.34
Ramachandran plot statistics	
Favored (%)	96.49
Allowed (%)	3.51
R.M.S. deviations	
Bond lengths (Å)	0.008
Bond angles (°)	0.90

Statistics for the plausible ligands

	R_{work} (%)[c]	R_{free} (%)[c]	Occupancy of the ligand[d]	Average B factor of the ligand (Å^2)	RSCC[e]
No ligand	18.31	21.80	-	-	-
CO_2	18.27	21.78	1.00	50.69	0.89
Glycerol	18.22	21.78	0.46	41.82	0.95
Carbonate	18.23	21.84	0.51	45.27	0.95

[a]Values in parentheses represent the highest resolution shell.
[b]R_{work} was calculated using 95% of the data included in the refinement and R_{free} the 5% excluded data.
[c]R_{work} and R_{free} were calculated by Phenix.Refine.
[d]The structure was refined by Phenix.Refine after the generation of each ligand.
[e]Real-space correlation coefficient was provided by wwPDB Validation Server.
